# An Assessment of the Public Health Risk Associated with Consumption of Imported Fish Based on the Intake of Essential and Harmful Elements

**DOI:** 10.3390/molecules30183836

**Published:** 2025-09-22

**Authors:** Agata Witczak, Artur Ciemniak, Beata Więcaszek, Sławomir Keszka, Mikołaj Protasowicki, Kamila Pokorska-Niewiada

**Affiliations:** 1 Department of Toxicology, Dairy Technology and Food Storage, West Pomeranian University of Technology in Szczecin, Papieża Pawła VI 3, 71-459 Szczecin, Poland; artur.ciemniak@zut.edu.pl (A.C.); protasowicki@zut.edu.pl (M.P.); 2 Department of Hydrobiology, Ichthyology and Reproductive Biotechnology, West Pomeranian University of Technology in Szczecin, Kazimierza Królewicza 4, 71-550 Szczecin, Poland; beata.wiecaszek@zut.edu.pl; 3 Border Veterinary Inspection Post in the Port of Szczecin, 70-606 Szczecin, Poland; keszka@gazeta.pl

**Keywords:** heavy metals, imported fish, local fish, bioaccumulation, potential health risk

## Abstract

Despite its many important health benefits, fish consumption is associated with a growing risk of toxicity due to increasing levels of environmental pollution. Therefore, this study compared the potential risks to human health associated with the consumption of imported fish and locally produced fish, which may be contaminated with toxic elements. A selection of the most commonly consumed fish in Poland, imported and domestic, was assayed for 11 trace elements in muscle tissue using ICP-AES, CV-AAS and GF-AAS. In general, the levels of toxic elements decreased according to the following sequence: As > Hg > Cd > Pb; however, the values of lead were slightly higher than those of cadmium in cod. All imported fish contained significantly more cadmium than the Polish species. Our assessment of EDI, THQ, TTHQ, TWI, PTMI and BMDL_01_ indicates that typical levels of consumption of fish do not pose a risk based on the assumed intake. The highest TTHQ was observed in tilapia, but it did not exceed 0.169. This was well below the acceptable value. Hence, the consumption of these fish does not appear to entail any non-carcinogenic or carcinogenic health risks. In addition, the estimated consumer risk parameters indicate no risk to consumer health in the short term; however, the presence of these elements may present a long-term hazard due to the potential for bioaccumulation. Continuous monitoring of trace element concentrations, especially toxic ones, is recommended for the protection of communities in both local and global contexts. Our findings provide a clearer picture of the health risk associated with the consumption of fish in the Polish market.

## 1. Introduction

For health-conscious consumers, one of the most desirable foods is fish. The chemical composition of raw fish is as follows: 0–0.5% carbohydrates, 16–21% protein, 1.2–1.5% minerals, 0.2–25% fat and 66–81% water. The muscle tissue is a source of long-chain omega-3 polyunsaturated fatty acids (n-3 LC-PUFA), amino acids, fat-soluble vitamins (A and D) and essential elements such as calcium, zinc and iron. As these compounds are frequently absent from diets leading to various health problems, even in developed countries [1,2,3], dieticians often recommend increasing the share of fish in the diet, both for preventive purposes and for treating diet-related diseases, such as diabetes [4,5]. Although fish are a valuable source of nutrients, they also absorb pollutants from their environment (polycyclic aromatic hydrocarbons, polychlorinated biphenyls, dioxins, microplastics, lead, cadmium, arsenic and mercury). Many of these pollutants demonstrate high persistence, biomagnification, bioaccumulation and non-biodegradability in food chains; as such, they can have considerable negative effects on aquatic ecosystems [3,6,7,8]. After consumption, the compounds acquired from fish can accumulate in the tissues and internal organs of the human body, with various positive and negative effects: The essential trace elements are needed for the activity of various enzymes, while the toxic elements can act as cofactors, initiators or promoters of various diseases. Considering the adverse, and even harmful, effects of certain elements on the human body, food safety organizations, such as the FAO, are introducing oral reference doses for most heavy metals and metalloids in food. The US EPA (Environmental Protection Agency) has developed an Integrated Risk Assessment program aimed at protecting human health from contamination from various sources [9,10,11]. Various factors are also introduced to determine consumer risk associated with consuming contaminated products (e.g., EDI, EWI, THQ, TTHQ, PTWI, BMDL, etc.). This complicates the analysis of results and contributes to inconsistent health risk assessments. Furthermore, combinations of elements can have synergistic or antagonistic effects, potentially resulting in a significantly higher impact [12,13,14].

Many authors have addressed the problem of trace element contamination in fish sold in local markets around the world [15,16,17,18]. However, the greatest concern is caused by imported fish, especially from Asia. Concerns primarily revolve around farming methods, feed quality and the use of substances that enhance fish production, which are banned in Europe. The most frequently mentioned species is the pangasius, whose farming is associated with the Mekong River, one of the most polluted rivers in the world [19,20]. In response to growing concerns about the safety of pangasius species originating from Vietnam, the European Commission in 2023, citing the applicable regulations [21], ruled that pangasius species originating from Vietnam did not pose a risk to food safety in the EU [22].

Geochemical properties and the cleanliness of ecosystems can influence the bioaccumulation of metals in fish [23,24,25]. No consistent pattern of metal content was observed among species or across locations.

One of the leading exporters of food and fish products is China. It provides approximately 260,000 tons of frozen fish fillets and fish products to Poland per year, placing the country among its leading European importers [26,27]. These fish include Nile tilapia (*Orechromis niloticus niloticus*), panga (*Pangasius pangasius*), Pacific cod (*Gadus macrocephalus*), pollock (*Pollachius virens*) and yellow-leafed sole (*Limanda aspera*). These imports are primarily driven by the lower cost of fish from the region.

The main aim of this study was to compare the levels of essential and harmful elements in samples of fish species imported to Poland with those caught in the country. All fish were frozen. The findings were used to determine the extent to which an edible portion of 100 g of fish product covers the recommended daily intake of essential trace elements. The study also aimed to assess the risk to human health associated with regular consumption of these species, which is an important consideration for consumer health.

## 2. Results and Discussion

Fish meat could be considered a cornerstone of a healthy human diet, with its consumption lowering the risk of coronary cardiac disease, hypertension and cancer. This is mainly due to its nutrient content, with the key elements presented in Table 1.

The lowest zinc content (0.94 mg/kg w.w.) was found in panga, and the highest (8.40 mg/kg w.w.) was found in rainbow trout (Table 1). The Polish trout, salmon and bream were found to contain significantly higher zinc levels than imported species (Appendix A).

Zinc is essential for the catalytic activity of many enzymes and plays an important role in enhancing immune function, protein and DNA synthesis, supporting wound healing and enabling cell signaling and division [28]. Both excess zinc and its deficiency in the diet can have serious health consequences. In Poland, the recommended intake is 8 mg/day for women and 11 mg/day for men [29]; similar values are recommended by the National Institutes of Health (NIH) [28]. Of all the fish analyzed, a portion of rainbow trout covers over 3% (for women) and almost 2.5% (for men) of these values (Table 2).

The nickel content in the analyzed fish did not exceed 0.37 mg/kg w.w., with significantly lower amounts being found in all imported fish compared to the Polish fish (Table 1). Nickel is not one of the most important elements in the human body, and its deficiency is rarely observed, as it is present in almost all types of food. However, lower levels can increase perinatal mortality and reduce growth rate and cause problems with iron absorption [30].

**Table 1:** Mean content (x ± SD) of trace elements in imported (A) and Polish (B) fish fillets (mg/kg w.w.). Comparison of the content of selected elements in the analyzed fish species with previous data.

	Nile Tilapia	Panga	Alaska Pollock	Hake	Yellowfin Sole	Pacific Cod	Flounder	Mackerel	Rainbow Trout	Salmon	Bream
	Mean, mean ± SD, or range of analyzed elements * (mg/kg w.w.)										
Zn	**3.89 ± 0.74** **2.59–5.38** 6.28 ± 0.39 [26] 58.3 ± 0.6 [31] 14.0 ** [32] 1.31–3.9 [33] 3.6–11.0 [34]	**2.08 ± 1.13** **0.94–5.92** 3.57 ± 0.02 [35]	**2.31 ± 0.65** **1.11–3.41** 3.44 ** [36]	**2.18 ± 0.30** **1.77–2.80** 3.34 ± 0.38 [37] 3.21 ** [36] 3.30 ** [38]	**2.72 ± 0.23** **2.34–3.03**	**3.19 ± 0.45** **2.63–3.72** 0.35 ± 0.01 [39]	**3.47 ± 0.32** **3.14–3.88** 2.41 ± 0.88 [40] 4.62 ± 0.71 [41]	**1.83 ± 0.54** **1.38–2.49** 0.27 ± 0.005 [39] 2.0 ± 0.3 [42] 8.74 ** [36] 8.95 ± 4.30 [40] 66.8 ± 1.6 [31]	**7.54 ± 1.00** **6.10–8.40** 5.6 ± 1.9 [26] 4.7–8.6 [43] 16.98 ** [44]	**4.86 ± 0.76** **4.18–5.58** 1.7–9.9 [43] 1.96 ± 0.04 [45] 3.20 ** [36]	**5.60 ± 0.54** **5.06–6.25** 2.3–3.5 [46]
Ni	**0.073 ± 0.06** **0.03–0.37** <0.01–0.07 [34] 5.75 ± 1.04 [26] 0.03 ± <0.01 [35]	**0.08 ± 0.03** **0.04–0.14** 0.10–0.17 [47]	**0.08** ± **0.03** **0.02–0.13** 0.090 ** [36]	**0.08 ± 0.06** **<LOD–0.17** <0.05 ** [38] 0.035 ** [36]	**0.09 ± 0.04** **0.05–0.14**	**0.10 ± 0.03** **0.10–0.11** 0.10 ± 0.01 [39]	**0.33 ± 0.02** **0.31–0.35** 0.02 ± 0.02 [41] 0.04 ± 0.06 [40]	**0.33 ± 0.03** **0.30–0.36** 0.070 ** [36] 0.82 ± 0.15 [42] 0.16 ± 0.12 [40] 0.03 ± 0.01 [39] 0.04 ± <0.01 [31]	**0.27 ± 0.02** **0.25–0.30** 2.38 ± 0.28 [26] 5.25 ** [44]	**0.33 ± 0.02** **0.31–0.36** 0.038 ** [36]	**0.32 ± 0.02** **0.29–0.34** <LOD–0.13 [46]
Fe	**1.84 ± 0.29** **1.04–2.29** <LOQ–91.5 [48] 2.4–17.0 [34] 28.0 ** [32]	**1.14 ± 0.39** **0.31–1.78** 9.35 ± 0.03 [35]	**1.45 ± 0.45** **0.96–2.58** 1.35 ** [36]	**1.42 ± 0.20** **1.06–1.70** 1.35 ** [36] 1.7 ** [38]	**1.32 ± 0.42** **0.85–1.77**	**1.53 ± 0.15** **1.31–1.62** 2.37 ± 0.10 [39]	**1.73 ± 0.28** **1.35–1.96** 3.52 ± 1.51 [40,41]	**1.61 ± 0.35** **1.17–1.90** 8.98 ** [36] 9.22 ± 0.11 [40,42] 3.16 ± 0.12 [39]	**1.57 ± 0.08** **1.48–1.67** 40.02 ** [44] 3.09–5.59 [43]	**1.48 ± 0.10** **1.35–1.60** 1.87 ** [36] 2.6 ± 0.21 [45] 2.46–11.90 [43]	**1.41 ± 0.10** **1.32–1.56** 1.3–1.5 [46]
Mn	**0.12 ± 0.03** **0.08–0.21** <LOQ–17.7 [48] 0.05–1.41 [33] 0.40 ± 0.02 [26] 1.0 ** [32]	**0.07 ± 0.03** **0.02–0.12** 0.14–0.20 [47] 0.22 ± 0.01 [35]	**0.05 ± 0.01** **0.02–0.07** 0.059 ** [36]	**0.05 ± 0.01** **0.02–0.07** 0.09 ** [38] 0.054 ** [36]	**0.06 ± 0.03** **0.02–0.08**	**0.06 ± 0.01** **0.05–0.07**	**0.12 ± 0.01** **0.11–0.14** 0.19 ± 0.12 [41] 0.07 ± 0.09 [40]	**0.07 ± 0.01** **0.06–0.07** 0.16 ** [36] 0.46 ± 0.11 [42] 0.03 ± 0.03 [40]	**0.08 ± 0.01** **0.07–0.09** 1.37 ± 0.12 [26] 0.25–0.36 [43]	**0.09 ± 0.01** **0.08–0.09** 0.110 ** [36] 0.14–0.87 [43]	**0.09 ± <0.01** **0.09–0.10** 0.11–0.39 [46]
Cr	**0.08 ± 0.03** **0.04–0.13** 0.007–0.8 [34] 3.6 ± 0.45 [39] 0.03 ± 0.06 [35] 2.79 ± 0.95 [49]	**0.09 ± 0.04** **0.04–0.17**	**0.08 ± 0.04** **0.02–0.15** 0.22 ** [36]	**0.09 ± 0.03** **0.04–0.13** 0.10 ** [38] 0.114 ** [36]	**0.08 ± 0.01** **0.06–0.10**	**0.08 ± 0.01** **0.07–0.09** 0.08 ** [50]	**0.1 ± <0.01** **0.09–0.10** 0.32 ± 0.04 [40]	**0.01 ± 0.01** **0.08–0.11** 0.18 ** [36] 0.87 ± 0.11 [42] 0.44 ± 0.04 [40] 0.03 ± <0.01 [31]	**0.13 ± 0.04** **0.10–0.17** 3.14 ** [44]	**0.13 ± <0.01** **0.012–0.013** 0.314 ** [36]	**0.11 ± <0.01** **0.012–0.014** 0.02–0.11 [46]
Cu	**0.15 ± 0.07** **0.06–0.33** <LOQ–42.9 [48] 0.23–0.47 [33] 0.2–9.0 [34] 1.6 ** [32]	**0.19 ± 0.04** **0.09–0.25** 0.16–0.23 [47] 0.64 ± 0.01 [35]	**0.20 ± 0.10** **0.07–0.39** 0.227 ** [36]	**0.13 ± 0.04** **0.08–0.19** 0.12 ± 0.04 [37] 0.16 ** [38] 0.136 ** [36]	**0.15 ± 0.02** **0.12–0.18**	**0.20 ± 0.01** **0.9–0.21** 0.127 ± 0.01 [39]	**0.19 ± 0.02** **0.02–0.22** 0.33 ± 0.16 [41]	**0.19 ± 0.02** **0.17–0.21** 1.03 ** [36] 0.72 ± 0.02 [42] 0.28 ± 0.003 [39]	**0.21 ± 0.01** **0.20–0.23** 2.06 ** [44] 0.19–0.55 [43]	**0.21 ± 0.01** **0.20–0.21** 0.580 ** [36] 0.98 ± 0.02 [45] 0.12–0.90 [43]	**0.21 ± <0.01** **0.20–0.21** 0.11–0.24 [46]
Al	**0.24 ± 0.05** **0.13–0.32** 0.3–8.7 [34]	**0.14 ± 0.03** **0.09–0.19**	**0.24 ± 0.09** **0.10–0.42** 2.53 ** [36]	**0.19 ± 0.06** **0.01–0.28** 0.834 ** [36]	**0.18 ± 0.03** **0.1–0.22**	**0.14 ± 0.02** **0.11–0.15**	**0.12 ± 0.01** **0.11–0.13** 1.72 ± 0.58 [41]	**0.13 ± 0.01** **0.12–0.14** 1.16 ** [36]	**0.11 ± 0.02** **0.08–0.13** 1.89–2.75 50]	**0.11 ± 0.03** **0.07–0.15** 0.529 ** [36] 0.23–3.72 [43]	**0.11 ± 0.01** **0.10–0.13**
Li	**0.03 ± 0.02** **0.01–0.08** 0.004–0.013 [34]	**0.04 ± 0.02** **0.01–0.08**	**0.03 ± 0.01** **0.01–0.05** 0.014 ** [36]	**0.02 ± 0.01** **0.01–0.04** 0.017 ** [36]	**0.04 ± 0.02** **0.02–0.07**	**0.04 ± 0.02** **0.02–0.06**	**0.05 ± 0.01** **0.04–0.06** 0.017 ± 0.007 [41]	**0.05 ± 0.02** **0.04–0.07** 0.018 ** [36]	**0.06 ± 0.01** **0.04–0.07**	**0.06 ± 0.01** **0.01–0.07** 0.005 ** [36]	**0.05 ± 0.01** **0.04–0.06** 0.01–0.05 [46]
As	**0.21 ± 0.08** **0.11–0.39** 0.01–0.06 [34] <0.001 ** [35]	**0.24 ± 0.08** **0.11–0.39** <0.017 ** [47]	**0.17 ± 0.05** **0.08–0.26**	**0.18 ± 0.05** **0.09–0.29** 7.86 ± 2.83 [37]	**0.23 ± 0.02** **0.20–0.27**	**0.16 ± 0.03** **0.12–0.19** 1.59 ** [50]	**0.21 ± 0.07** **0.12–0.28** 5.29 ± 1.87 [40]	**0.13 ± 0.03** **0.10–0.16** 2.51 ± 0.75 [40] <LOD [31]	**0.14 ± 0.01** **0.13–0.15**	**0.16 ± 0.05** **0.09–0.22**	**0.10 ± 0.03** **0.05–0.12** 0.0–0.3 [51]
Pb	**0.026 ± 0.009** **0.013–0.047** <LOQ–0.3 [48] 0.34–2.7 [33] 0.001–0.041 [34] 0.094 ± 0.055 [35] 3.49 ± 1.86 [49]	**0.024 ± 0.010** **0.010–0.014** 0.12–0.17 [47]	**0.019 ± 0.005** **0.011–0.031** 0.010 ** [36]	**0.017 ± 0.004** **0.012–0.024** 0.016 ± 0.005 [37] 0.002 ± 0.001 [52] 0.010 ** [36]	**0.013 ± 0.04** **0.007–0.016**	**0.011 ± 0.002** **0.010–0.014** 0.024 ** [50] 0.202 ± 0.01 [39]	**0.010 ± 0.003** **0.125–0.276** 0.053 ± 0.052 [41] 0.021 ± 0.059 [40]	**0.008 ± 0.003** **0.103–0.163** 0.009 ** [36] 0.122 ± 0.021 [42] 0.075 ± 0.024 [40] 0.457 ± 0.016 [39] 0.046 ± 0.004 [31]	**0.009 ± 0.002** **0.129–0.152** <LOD [44] 0.017–0.035 [43]	**0.010 ± 0.003** **0.093–0.222** 0.005 ** [36] <0.01–0.02 [43]	**0.011 ± 0.004** **0.053–0.123** 0–0.115 [7,51] 0–0.02 ** [46]
Cd	**0.040 ± 0.013** **0.011–0.073** 0.09–1.17 [33] <0.0025 [34] 0.051 ± 0.004 [26] 0.003 ± <0.001 [31] 0.40 ± 0.01 [49]	**0.043 ± 0.011** **0.026–0.072** <0.006 [47]	**0.032 ± 0.007** **0.020–0.048**	**0.036 ± 0.008** **0.021–0.048** 0.01 ± 0.01 [37] 0.003 ± 0.001 [52]	**0.032 ± 0.010** **0.013–0.045**	**0.010 ± 0.006** **0.005–0.018** <0.002 [53] 0.030 ± 0.001 [39]	**0.011 ± 0.002** **0.008–0.012** 0.009 ± 0.006 [41] 0.002 ± <0.001 [40]	**0.011 ± 0.004** **0.006–0.016** 0.007 ± 0.002 [42] 0.003 ± 0.003 [40] 0.488 ± 0.012 [39] <LOD [31]	**0.013 ± 0.004** **0.008–0.017** 0.007 ± 0.001 [26] <LOD [44] <LOD–0.012 [43]	**0.020 ± 0.006** **0.012–0.027** <LOD–0.008 [43]	**0.019 ± 0.004** **0.016–0.024** <LOD–0.060 [7,52] <LOD–0.003 [46]
Hg	**0.055** ± **0.082** **0.025–0.058** <LOD–0.16 [48] 0.001–0.16 [34]	**0.054 ± 0.010** **0.031–0.070** 0.10–0.69 [54] 0.002–0.018 [47]	**0.043 ± 0.008** **0.028–0.058** 0.037 ** [55]	**0.040 ± 0.011** **0.031–0.070** 0.44 ± 0.06 [56] 0.023 ± 0.002 [52] 0.041 ** [55]	**0.045 ± 0.005** **0.036–0.051**	**0.031 ± 0.009** **0.022–0.043** 0.049 ** [55] 0.128 ** [50]	**0.058 ± 0.002** **0.055–0.060** 0.036 ± 0.017 [41] 0.016 ± 0.017 [40] 0.056 ** [55]	**0.037 ± 0.004** **0.033–0.040** 0.152 ± 0.096 [40] 0.058 ** [55]	**0.035 ± 0.003** **0.033–0.038** 0.010 ** [55]	**0.041 ± 0.002** **0.039–0.042**	**0.029 ± 0.007** **0.023–0.039** <LOD–0.391 [7,51] <LOD–0.01 [46]

Bold: The present findings, * The given value depends on the information given in the manuscripts of other authors, ** No information on standard deviation.

**Table 2:** Coverage of the daily requirement for micronutrients (%) according to Polish regulations [29].

	Zn Mean ± SD (Range)		Fe Mean ± SD (Range)		Mn Mean ± SD (Range)		Cu Mean ± SD (Range)
	Women	Men	Women	Men	Women	Men	Men/Women
Polish Recommendations	RDA 8.0 mg/day	RDA 11.0 mg/day	RDA 18.0 mg/day	RDA 10.0 mg/day	AI 1.8 mg/day	AI 2.3 mg/day	RDA 0.9 mg/day
Nile tilapia	1.7 ± 0.3 (1.1–2.3)	1.2 ± 0.2 (0.8–1.7)	0.36 ± 0.06 (0.20–0.44)	0.64 ± 0.10 (0.36–0.80)	0.23 ± 0.06 (0.17–0.41)	0.18 ± 0.05 (0.13–0.32)	0.58 ± 0.28 (0.24–1.27)
Pacific cod	1.4 ± 0.2 (1.1–1.6)	1.0 ± 0.1 (0.8–1.2)	0.30 ± 0.03 (0.25–0.31)	0.53 ± 0.05 (0.46–0.56)	0.12 ± 0.02 (0.10–0.14)	0.09 ± 0.02 (0.08–0.11)	0.78 ± 0.05 (0.72–0.82)
Panga	0.9 ± 0.5 (0.4–2.6)	0.7 ± 0.4 (0.3–1.9)	0.22 ± 0.08 (0.06–0.34)	0.40 ± 0.14 (0.11–0.62)	0.14 ± 0.06 (0.03–0.23)	0.11 ± 0.05 (0.03–0.18)	0.72 ± 0.15 (0.36–0.96)
Alaska pollock	1.0 ± 0.3 (0.5–1.5)	0.7 ± 0.2 (0.4–1.1)	0.28 ± 0.09 (0.19–0.50)	0.50 ± 0.16 (0.33–0.90)	0.10 ± 0.02 (0.04–0.14)	0.08 ± 0.02 (0.03–0.11)	0.76 ± 0.36 (0.26–1.49)
Yellowfin sole	1.2 ± 0.1 (1.0–1.3)	0.9 ± 0.1 (0.7–1.0)	0.26 ± 0.08 (0.16–0.34)	0.46 ± 0.15 (0.29–0.61)	0.11 ± 0.06 (0.04–0.16)	0.08 ± 0.04 (0.03–0.13)	0.57 ± 0.08 (0.45–0.69)
North-Pacific hake	0.9 ± 0.1 (0.8–1.2)	0.7 ± 0.1 (0.6–0.9)	0.27 ± 0.04 (0.21–0.33)	0.49 ± 0.07 (0.37–0.59)	0.10 ± 0.03 (0.04–0.13)	0.08 ± 0.02 (0.03–0.10)	0.49 ± 0.15 (0.32–0.72)
Flounder	1.5 ± 0.1 (1.7–1.7)	1.1 ± 0.1 (1.0–1.2)	0.33 ± 0.05 (0.26–0.38)	0.60 ± 0.10 (0.47–0.68)	0.22 ± 0.03 (0.21–0.26)	0.17 ± 0.02 (0.16–0.21)	0.74 ± 0.07 (0.67–0.84)
Mackerel	0.8 ± 0.2 (0.6–1.1)	0.6 ± 0.2 (0.4–0.8)	0.31 ± 0.07 (0.23–0.37)	0.56 ± 0.12 (0.41–0.66)	0.13 ± 0.01 (0.12–0.14)	0.10 ± 0.01 (0.10–0.11)	0.73 ± 0.06 (0.67–0.81)
Rainbow trout	3.3 ± 0.4 (2.7–3.7)	2.4 ± 0.3 (1.9–2.7)	0.30 ± 0.02 (0.29–0.32)	0.55 ± 0.03 (0.52–0.58)	0.16 ± 0.01 (0.14–0.16)	0.12 ± 0.01 (0.11–0.13)	0.83 ± 0.05 (0.79–0.90)
Salmon	2.1 ± 0.3 (1.8–2.4)	1.5 ± 0.2 (1.3–1.8)	0.29 ± 0.02 (0.26–0.31)	0.52 ± 0.03 (0.47–0.56)	0.17 ± 0.01 (0.17–0.18)	0.13 ± 0.01 (0.13–0.14)	0.79 ± 0.02 (0.77–0.81)
Bream	2.4 ± 0.2 (2.2–2.7)	1.8 ± 0.2 (1.6–2.0)	0.27 ± 0.02 (0.25–0.30)	0.49 ± 0.04 (0.46–0.54)	0.18 ± <0.01 (0.17–0.18)	0.14 ± 0.01 (0.14–0.14)	0.79 ± 0.02 (0.78–0.81)

SD—standard deviation; RDA—recommended dietary allowance [29]; AI—adequate intake [29].

The iron content ranged from 0.31 to 2.29 mg/kg w.w. and did not differ significantly between the analyzed fish species (Table 1, Appendix A). Iron is an essential component of hemoglobin and myoglobin and occurs in the diet as heme and non-heme forms; the heme form is found only in meat and seafood [29]. Our data indicate that a portion of the tested fish will cover about 0.3% of the iron requirement in women and a little over 0.6% in men (Table 2).

Significantly higher levels of manganese were observed in tilapia and flounder than in the other species (Table 1). Manganese is a cofactor of many enzymes, including manganese superoxide dismutase, pyruvate carboxylase and arginase [53]. Consuming a portion of these fish will cover approximately 0.2% of the requirement in both women and men (Table 2).

Additionally, significantly higher amounts of chromium were found in rainbow trout than in the other species (Table 1). Chromium takes part in the metabolism of carbohydrates, lipids and proteins by enhancing the action of insulin [57].

The copper content varied widely between 0.062 and 0.386 mg/kg w.w. (Table 1), with no statistically significant differences observed between species. Copper is a cofactor of various enzymes involved, inter alia, in energy production, iron metabolism, neuropeptide activation, connective tissue synthesis and neurotransmitter synthesis [58].

The aluminum content ranged from 0.072 to 0.416 mg/kg, with two imported species (tilapia and pollock) containing significantly more aluminum than the others (Table 1). The highest lithium content was found in salmon, and the lowest in hake; the levels in both species differed significantly (*p* < 0.05) from those found in the others (Table 1).

The concentration of arsenic in Nile tilapia sold in Bangkok markets was 0.50 mg/kg [59], while in this study, it was half as high. For arsenic and mercury, only single significant relationships were found between species.

Fish are a potential source of mercury in the human diet. Therefore, the European Commission has set the maximum level of this element in muscle at 0.50 mg/kg [21]. This study indicates that the tested fish are safe in this regard. Considering the safety of sensitive individuals—children, pregnant women, breastfeeding mothers and the elderly—special attention should be paid to elevated levels of this element, even if they are within the permissible range. However, there are reports by Rodriguez et al. [54] and Ferrantelli et al. [60] indicating elevated mercury levels in pangasius fillets. Moreover, Ferantelli et al. [60] indicate that elevated mercury levels in pangasius may result from the origin, breeding, feeding, storage and processing of these fish. They also note that the quality of water used during fillet freezing may contribute to the presence of high Hg levels.

For lead, significantly higher concentrations were observed in the tissues of pollock and pangasius (Appendix A). Similarly, lead accumulation in Baltic cod and rainbow trout in the present study was almost 10 times higher than the results reported by Staszowska et al. [61] for the same fish species.

Despite the low levels of toxic elements found in local fish in this study, significant levels of lead have been reported in rainbow trout (from Polish farms) and cod from the Baltic Sea [18]. This confirms the need for ongoing monitoring of fish contamination with toxic elements.

All imported fish contained significantly more cadmium than the Polish species. While toxic elements were found in the analyzed fish, their concentrations were much lower than the maximum limits given by the FAO/WHO and the European Union for heavy metals in fish [10,21,28]. Cadmium levels in bream from Slovakia were three times lower (0.0007 mg/kg) than those analyzed in this study, while mercury concentrations in fish from Slovakia were five times higher (0.155 mg/kg). It is important to note that this is the Zemplínska Šírava reservoir, one of the most polluted in Europe, due in part to the presence of polychlorinated biphenyls [62].

In general, the levels of toxic elements decreased according to the following sequence: As > Hg > Cd > Pb; however, the values of lead were slightly higher than those of cadmium in cod (Table 1). The differences in metal accumulation among species could be related to their unique physiology and ecological niche [63].

Based on the results, it can be concluded that the diversity of origins of fish used for human consumption significantly influences their composition, including heavy metal content. The differences in the chemical composition of the meat of the above-mentioned fish species are certainly due to their diverse origins. Breeding conditions vary dramatically, depending on the species’ sensitivity to water parameters.

It is widely believed that eating fish can play a significant part in maintaining good health. Fish meat consists of high-quality protein containing all the essential amino acids, as well as a number of polyunsaturated omega-3 fatty acids, vitamins and minerals, particularly zinc and iron. However, many of these essential trace elements are only available in the head, bones and viscera, and hence are only acquired when consuming small fish whole [64,65]. Fish are generally consumed in insufficient quantities to ensure a significant share of the daily requirement for trace elements (Table 2). Current recommendations for fish consumption vary across Europe and range from 100 to 482 g/person/week for adults [3,66,67]. However, most countries recommend higher fish consumption, i.e., at least two portions per week. The Polish Institute of Food and Nutrition [68] and the EUMOFA report [69] that Poland is characterized by low fish consumption among the European Union countries. Alarmingly, young people in Poland are reported to consume only 13.4 g of fish per week, with the main reason being a fear of contamination [67].

**Figure 1:**
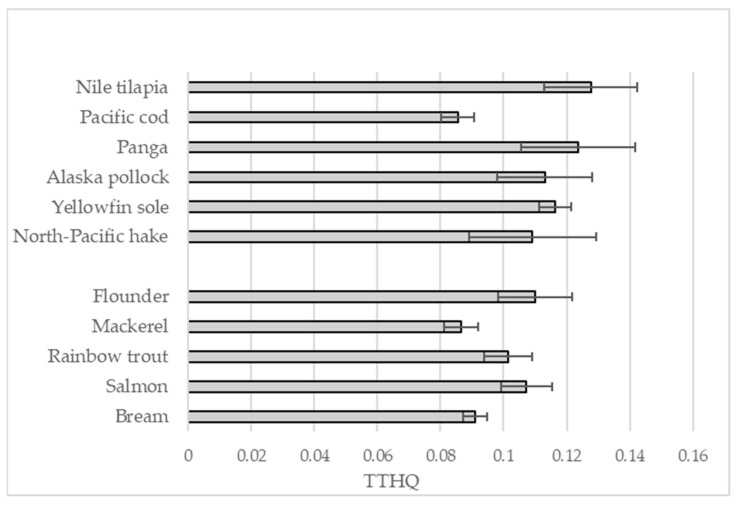
Total target hazard quotient of all samples.

Our assessment of EDI, THQ, TTHQ, TWI, PTMI and BMDL_01_ indicates that typical levels of consumption of fish do not pose a risk based on the assumed intake (Table 3 and Table 4, Figure 1). Previous studies based on the same indicators have also found there to be negligible risk, e.g., in Brazil [70] and in Iran (Hg, Cd and Pb) [71]. A study of fish from Lake Temsah (Suez Canal region, Egypt) did not indicate any significant potential risk to health (EDI < 1) [8], nor did a study of the consumption of rainbow trout from Samsun fish markets (Turkey) (TTHQ = 0.084) [72]. However, there are regions where fish consumption may pose a risk of contamination with toxic elements. An Iranian study indicated a risk of mercury, cadmium and lead poisoning among fish caught from the Caspian Sea region (TTHQ = 0.975), and mercury poisoning in fish caught in the Persian Gulf and Oman (THQ = 1.347) [71]. It has also been found that fish commonly purchased in shops in Nigeria, Osun State, may contain toxic levels of cadmium for both adults (EDI = 1.708) and children (EDI = 7.471) [73].

Information about food potentially contaminated with toxic elements should be provided to consumers to prevent negative health consequences. Government authorities responsible for food safety should regularly monitor levels of toxic element contamination in newly introduced food products. Following European Commission guidelines, special attention should also be paid to groups of people at increased risk of poisoning from toxic elements. This includes children, pregnant women, breastfeeding mothers and the elderly. Expanding information campaigns and preventing misinformation about fish from foreign markets is also recommended. It should be emphasized that knowledge of the location of the waters from which the fish were caught, and the environmental conditions prevailing in those locations is crucial. It is also important to pay attention to the characteristics of the fish, its diet and lifespan, as toxic elements have the potential to bioaccumulate and bioconcentrate.

## 3. Materials and Methods

### 3.1. Characteristics of the Tested Material

The research material included eleven species of fish. Six species were imported from China and Vietnam, viz. Nile tilapia (*Orechromis niloticus niloticus*), panga (*Pangasius pangasius*), Alaska pollock (*Gadus chalcogrammus*), North-Pacific hake (*Merluccius productus*), yellowfin sole (*Limanda aspera*) and Pacific cod (*Gadus macrocephalus*). In addition, five species were purchased from various batches in retail trade in Poland, Szczecin, viz. flounder (*Platichthys flesus*), mackerel (*Scomber scombrus*), rainbow trout (*Oncorhynchus mykiss*), salmon (*Salmo salar*) and bream (*Abramis brama*). Individual batches of fish (the number of batches for each fish species and the number of samples are in Table 5) came from different producers (the list of producers is known to the authors). To ensure representativeness, the batches of fish for testing were selected randomly. Each batch weighed about 2.5 kg. A total of 790 samples, 10 from each batch, were tested. The characteristics of the collected material are presented in Table 5.

All fish, both domestic and foreign, were purchased gutted. They were not euthanized in the authors’ laboratory. Therefore, ethics committee approval was not required.

### 3.2. Determination of Elements

Muscle samples weighing 1 g ± 0.001 g were ground and mineralized in a CEM MDS-2000 microwave oven (SpectraLab Scientific Inc., Markham, ON, Canada) with the addition of 3 mL of EMSURE, 65% nitric acid (Merck KGaA, Darmstadt, Germany). After mineralization, the samples were filtered and transferred to polyethylene bottles using deionized water. The bottles were weighed and filled with deionized water (0.05 μS cm^−1^; Barnstead™ Gen-Pure™ Pro, Thermo Scientific, Erlangen, Germany) to a mass of 25.0 g ± 0.1 g. The levels of Zn, Ni, Fe, Mn, Cr, Cu, Al and Li were determined using the ICP-AES method (Jobin Yvon JY-24, Longjumeau, France) fitted with a Meinhard (Singapore) TR 50-C1 nebulizer. The generator operated with an output power of 1000 W; frequency, 40.68 MHz; and the plasma gas and the nebulizer gas were argon with flow rates of 12.0 L/min., nebulizer flow rate of 0.7 L/min and sample solution flow rate of 1.0 mL/min.

The arsenic content was also determined with an ICP-AES using the hydride generation system. The solutions of the analyzed samples and the NaBH_4_ and HCl reagents were introduced simultaneously using two dual-channel peristaltic pumps [74].

The following wavelengths were used for the analyses (nm): Zn—213.9; Ni—231.6; Fe—238.2; Mn—257.6; Cr—357.9; Cu—324.7; Al—396.2; Li—670.8 and As—193.8.

Cadmium and lead contents were determined by GF-AAS with Zeeman background correction (4110 ZL Perkin Elmer, Waltham, MA, USA). While in the furnace, the samples underwent five temperature stages from 110 to 2500 °C, with atomization at 1600 °C (Pb) and 1550 °C (Cd). The following settings were applied: slit width, 0.7 mm; lamp current, 10 mA (Pb) and 4 mA (Cd).

Mercury (Hg) content was determined by CV-AAS using a Bacharach Coleman MAS-50 mercury analyzer (Bacharach Inc., Pittsburgh, PA, USA) at a wavelength of λ = 253.7 nm. All samples were analyzed in three analytical replicates.

The precision of the analytical method was verified with the MODAS-3 certified reference material (Consortium “MODAS”, Warsaw, Poland). The quality was verified based on LOD, LOQ and recovery (Table 6).

### 3.3. Evaluation of Element Concentrations in Relation to Nutrient Requirement

To determine the extent to which the daily consumption of the analyzed fish met the demand for the studied nutrients, the findings were compared with the recommended values for the Polish population. These are as follows: zinc and iron—8 mg/day for women and 11 mg/day for men; copper—0.9 mg/day, lithium—1 mg/day, manganese (AI)—1.8 mg/day for women and 2.3 mg/day for men [29]. Referring to the most recent Statistical Yearbook of Agriculture, it was assumed that an adult weighing 70 kg consumes 13.4 g/day of fish [75].

### 3.4. Assessment of Concentrations of Harmful Elements in Relation to Standards and Risk Factors

To assess the possible health effects resulting from the consumption of imported fish, the EDI, EWI and THQ (Equations (1)–(3)) of the component nutrients were calculated [76,77]. These indicators determine potential non-cancer risks to human health. A THQ score above 1 indicates potential systemic effects. If the EDI ≥ RfD, the exposed human population will experience a health risk.

**EDI** (Estimated Daily Intake, Equation (1))

(1) $$EDI=\frac{MS\times C}{BW} [\frac{\mu g/kg\;bw}{day}]$$

MS—the daily food ingestion rate in grams per day (13.4 g/day) [75]; C—fresh weight concentration of trace elements in fish (mg/kg); BW—reference body weight (70 kg).

**THQ** (Target Hazard Quotient, Equation (2))

(2) $$THQ=\frac{EF\times ED\times MS\times C}{RfD\times BW\times AT}\times 10^{-3}$$

EF—exposure frequency to trace elements (365 days/year); ED—exposure duration (70 years); MS—food ingestion rate, 13.4 g/day [75]; C—concentration of trace element in fish (mg/kg); RfD—oral reference dose of trace element (mg/kg BW/day) (Zn = 0.3; Ni = 0.02; Fe = 0.7; Mn = 0.14; Cr = 0.003; Cu = 0.04; Li = 0.02; Cd = 0.001) [76,78]; BW—reference body weight of 70 kg; AT—averaged exposure time to non-carcinogenic trace elements (365 days × 70 years).

**TTHQ** (Total Target Hazard Quotient), i.e., total THQ of all elements analyzed (Equation (3))

(3) $$\begin{array}{ll} TTHQ= & THQ\left(Zn\right)+THQ\left(Ni\right)+THQ\left(Fe\right)+THQ\left(Mn\right)+THQ\left(Cr\right)+\\ & THQ\left(Cu\right)+THQ\left(Al\right)+THQ\left(Li\right)+THQ\left(As\right)+THQ\left(Cd\right)+\\ & THQ(Hg) \end{array}$$

**TWI** (Tolerable Weekly Intake, Equation (4))

(4) $$\%\;TWI=\frac{EDI\times 7\times 100}{TWI}$$

where the TWI value for Cd = 2.5 μg/kg BW per week.

Due to the damage caused to human health, the committee JECFA [79] established a PTMI for cadmium of 25 μg/kg BW (Equation (5)).

**PTMI** (Provisional Tolerable Monthly Intake, Equation (5))

(5) $$\%\;PTMI=\frac{EDI\times 30\times 100}{PTMI}$$

where the PTMI value for cadmium = 25 μg/kg BW per month [16]

The committee JECFA [79] also determined a BMDL value for Pb of BMDL_01_—1.5 μg/kg/BW per day for adult consumers (Equation (6)).

**BMDL** (Benchmark Dose Lower Confidence Limit, Equation (6))

(6) $${\%BMDL}_{0.1}=\frac{EDI\times 100}{{BMDL}_{0.1}}$$

where the BMDL_0.1_ for Pb, for effects on systolic blood pressure in adults, =1.50 μg/kg BW per day.

### 3.5. Statistical Analysis

The descriptive statistics are presented as mean ± SD (in tables). All statistical analyses were performed using the Statistica 13.0 software package (Statsoft, Cracow, Poland). Analysis of variance with ANOVA test was preceded by Levene’s test of homogeneity of variance and the Kologorov–Smirnov test of distribution normality. The arithmetical mean, standard deviation, and the significance of differences (Duncan’s test) were calculated. The level of significance was assumed to be α = 0.05. The correlation between the parameters was determined using Pearson’s correlation statistics.

## 4. Conclusions

Following WHO and EU guidelines, the authors suggest that the prevailing belief in Poland that some imported fish are contaminated with heavy metals may be unfounded. Although some differences in mineral content between imported and local fish were observed in this study, all metals appear to be present at safe levels, including toxic metals. On the other hand, although the risk factors are <1, the relatively higher levels of cadmium in imported species merit explicit discussion as a potential long-term risk. Similarly, mercury and arsenic levels in fish muscle vary significantly depending on the catch location and species. Disseminating such information could influence consumer choices and contribute to a greater interest in local fish species. Furthermore, it would encourage caution when trying local delicacies in areas with higher levels of environmental pollution. However, it should be noted that the results obtained are only partially conclusive, as the conclusions are based solely on information provided by vendors (catchment area). To assess the actual risk of metal contamination in fish, other factors must be taken into account, such as the age and sex of the fish, living conditions, pollution of the aquatic environment and diet, which also play a role in the bioaccumulation of elements.

## Figures and Tables

**Table 3:** Potential human health risk assessment (EDI, THQ).

EDI													
**Fish Species**		**Zn**	**Ni**	**Fe**	**Mn**	**Cr**	**Cu**	**Al**	**Li**	**As**	**Pb**	**Cd**	**Hg**
Nile tilapia	x	1.93	0.04	0.92	0.059	0.039	0.075	0.121	0.017	0.104	0.013	0.020	0.021
Pacific cod	x	1.58	0.05	0.76	0.031	0.041	0.100	0.067	0.018	0.079	0.005	0.005	0.015
Panga	x	1.03	0.04	0.57	0.036	0.045	0.093	0.068	0.019	0.120	0.012	0.022	0.027
Alaska pollock	x	1.15	0.04	0.72	0.026	0.040	0.097	0.120	0.015	0.083	0.009	0.016	0.021
Yellowfin sole	x	1.35	0.05	0.66	0.027	0.040	0.073	0.090	0.018	0.115	0.006	0.016	0.022
North-Pacific hake	x	1.08	0.04	0.71	0.026	0.045	0.062	0.092	0.010	0.088	0.009	0.018	0.020
Flounder	x	1.72	0.17	0.86	0.057	0.048	0.095	0.059	0.026	0.105	0.005	0.005	0.029
Mackerel	x	0.91	0.17	0.80	0.034	0.047	0.094	0.063	0.026	0.062	0.004	0.005	0.018
Rainbow trout	x	3.75	0.13	0.78	0.040	0.065	0.106	0.054	0.028	0.070	0.005	0.006	0.017
Salmon	x	2.41	0.16	0.74	0.044	0.063	0.102	0.056	0.032	0.080	0.005	0.010	0.020
Bream	x	2.78	0.16	0.70	0.046	0.056	0.102	0.056	0.024	0.048	0.006	0.010	0.014
**THQ**													
**Fish species**		**Zn**	**Ni**	**Fe**	**Mn**	**Cr**	**Cu**	**Al**	**Li**	**As**	**Pb**	**Cd**	**Hg**
Nile tilapia	x	0.006	0.002	0.001	<0.001	0.013	0.002	0.030	0.001	0.035		0.020	0.013
Pacific cod	x	0.005	0.003	0.001	<0.001	0.014	0.002	0.017	0.001	0.026		0.005	0.010
Panga	x	0.003	0.002	0.001	<0.001	0.015	0.002	0.017	0.001	0.040		0.022	0.017
Alaska pollock	x	0.004	0.002	0.001	<0.001	0.013	0.002	0.030	0.001	0.028		0.016	0.013
Yellowfin sole	x	0.004	0.002	0.001	<0.001	0.013	0.002	0.023	0.001	0.038		0.016	0.014
North-Pacific hake	x	0.004	0.002	0.001	<0.001	0.015	0.002	0.023	0.001	0.029		0.018	0.012
Flounder	x	0.006	0.008	0.001	<0.001	0.016	0.002	0.015	0.001	0.035		0.005	0.018
Mackerel	x	0.003	0.008	0.001	<0.001	0.016	0.002	0.016	0.001	0.021		0.005	0.011
Rainbow trout	x	0.012	0.007	0.001	<0.001	0.022	0.003	0.014	0.001	0.023		0.006	0.011
Salmon	x	0.008	0.008	0.001	<0.001	0.021	0.003	0.014	0.002	0.027		0.010	0.013
Bream	x	0.009	0.008	0.001	<0.001	0.019	0.003	0.014	0.001	0.016		0.010	0.009

**Table 4:** Coverage of % TWI, % PTMI, % BMDL_0.1_.

	Cd	Cd	Pb
Fish Species	% TWI x ± SD	% PTMI x ± SD	% BMDL_0.1_ x ± SD
Nile tilapia	5.53 ± 1.82	2.37 ± 0.78	0.87 ± 0.30
Pacific cod	1.39 ± 0.87	0.59 ± 0.37	0.36 ± 0.06
Panga	6.05 ± 1.52	2.59 ± 0.65	0.78 ± 0.33
Alaska pollock	4.48 ± 1.00	1.92 ± 0.43	0.62 ± 0.16
Yellowfin sole	4.43 ± 1.39	1.90 ± 0.60	0.43 ± 0.12
North-Pacific hake	5.04 ± 1.09	2.16 ± 0.47	0.57 ± 0.12
Flounder	1.48 ± 0.26	0.64 ± 0.11	0.34 ± 0.09
Mackerel	1.47 ± 0.55	0.63 ± 0.24	0.27 ± 0.09
Rainbow trout	1.76 ± 0.54	0.75 ± 0.23	0.30 ± 0.07
Salmon	2.74 ± 0.84	1.17 ± 0.36	0.33 ± 0.08
Bream	2.68 ± 0.50	1.15 ± 0.21	0.37 ± 0.12

**Table 5:** Characteristics of the tested material.

Species	Amount of Material Tested	Amount of Analysed Samples	Country of Origin	Fat Content % ^a^	Dry Weight % ^a^
Nile tilapia	18	180	China	1.39 ± 0.47	18.39 ± 1.71
Panga	27	270	Vietnam	1.09 ± 0.73	10.36 ± 2.19
Alaska Pollock	12	120	China	0.67 ± 0.09	8.60 ± 1.42
North-Pacific hake	3	12	China	2.05 ± 0.12	6.81 ± 1.02
Yellowfin sole	9	90	China	1.07 ± 0.17	11.25 ± 1.13
Pacific cod	13	130	China	0.50 ± 0.06	16.79 ± 0.29
Flounder	10	100	Poland	2.08 ± 0.05	20.08 ± 1.22
Mackerel	30	300	Poland	13.01 ± 0.11	27.14 ± 2.06
Rainbow trout	12	120	Poland	4.7 ± 0.12	24.03 ± 1.33
Salmon	8	80	Poland	13.5 ± 0.32	18.5 ± 0.1.12
Bream	22	220	Poland	3.5 ± 0.32	17.51 ± 0.98

^a^ Arithmetic mean ± standard deviation.

**Table 6:** LOD, LOQ values and recovery of analyzed samples.

Trace Element	LOD µg/kg	LOQ µg/kg	Recovery %	Trace Element	LOD µg/kg	LOQ µg/kg	Recovery %
**Zn**	20.4	62.8	98.8	**Al**	5.5	18.8	96.9
**Ni**	1.4	4.1	96.9	**Li**	1.3	4.0	97.6
**Fe**	19.8	60.4	98.4	**As**	0.3	1.0	95.9
**Mn**	1.2	3.9	97.7	**Pb**	0.8	3.1	99.2
**Cr**	1.4	4.5	98.2	**Cd**	0.31	1.1	98.9
**Cu**	8.6	26.1	99.1	**Hg**	1.6	5.2	96.7

## Data Availability

Data are contained within the article.

## Abbreviations

The following abbreviations are used in this manuscript:

AI	Adequate Intake
BMDL	Benchmark Dose Lower Confidence Limit
CV-AAS	Cold Vapor Atomic Absorption Spectrometry
EDI	Estimated Daily Intake
EUMOFA	European Observatory on the Market in Fisheries and Aquaculture
FAO/WHO	Food and Agriculture Organization (FAO) and the World Health Organization (WHO)
GF-AAS	Graphite Furnace–Atomic Absorption Spectrometry
ICP-AES	Inductively Coupled Plasma Atomic Emission Spectrophotometry
JECFA	Joint FAO/WHO Expert Committee on Food Additives
LOD	Limits of Detection
LOQ	Limits of Quantification
NaBH_4_	Sodium borohydride
NIH	National Institutes of Health
PTMI	Provisional Tolerable Monthly Intake
RDA	Recommended Dietary Allowance
RfD	Reference Dose
SD	Standard Deviation
THQ	Target Hazard Quotient
TTHQ	Total Target Hazard Quotient
TWI	Tolerable Weekly Intake
w.w.	Wet weight

## Appendix A

**Table A1:** Significant differences (marked in bold) in the content of elements between individual fish species (*p* < 0.05).

Zn Fish Species	Nile Tilapia	Pacific Cod	Panga	Alaska Pollock	Yellowfin Sole	North-Pacific Hake	Flounder	Mackerel	Rainbow Trout	Salmon
Pacific cod	0.1420									
Panga	**0.0002**	**0.0252**								
Alaska pollock	**0.0010**	0.0642	0.6285							
Yellowfin sole	**0.0152**	0.2933	0.1992	0.3674						
North-Pacific hake	**0.0004**	**0.0382**	0.8241	0.7666	0.2612					
Flounder	0.3530	0.5291	**0.0048**	**0.0164**	0.1127	**0.0083**				
Mackerel	**0.0000**	**0.0062**	0.5862	0.3394	0.0799	0.4742	**0.0008**			
Rainbow trout	**0.0000**	**0.0000**	**0.0000**	**0.0000**	**0.0000**	**0.0000**	**0.0000**	**0.0000**		
Salmon	**0.0301**	**0.0004**	**0.0000**	**0.0000**	**0.0000**	**0.0000**	**0.0028**	**0.0000**	**0.0000**	
Bream	**0.0002**	**0.0000**	**0.0000**	**0.0000**	**0.0000**	**0.0000**	**0.0000**	**0.0000**	**0.0000**	0.0975
**Ni** Fish species	Nile tilapia	Pacific cod	Panga	Alaska pollock	Yellowfin sole	North-Pacific hake	Flounder	Mackerel	Rainbow trout	Salmon
Pacific cod	0.3042									
Panga	0.9050	0.3355								
Alaska pollock	0.8991	0.3489	0.9988							
Yellowfin sole	0.4953	0.6777	0.5403	0.5566						
North-Pacific hake	0.8159	0.3799	0.8942	0.9004	0.6007					
Flounder	**0.0000**	**0.0000**	**0.0000**	**0.0000**	**0.0000**	**0.0000**				
Mackerel	**0.0000**	**0.0000**	**0.0000**	**0.0000**	**0.0000**	**0.0000**	0.9770			
Rainbow trout	**0.0000**	**0.0000**	**0.0000**	**0.0000**	**0.0000**	**0.0000**	**0.0098**	**0.0091**		
Salmon	**0.0000**	**0.0000**	**0.0000**	**0.0000**	**0.0000**	**0.0000**	0.9360	0.9542	**0.0087**	
Bream	**0.0000**	**0.0000**	**0.0000**	**0.0000**	**0.0000**	**0.0000**	0.4880	0.4862	**0.0409**	0.4910
**Fe** Fish species	Nile tilapia	Pacific cod	Panga	Alaska pollock	Yellowfin sole	North-Pacific hake	Flounder	Mackerel	Rainbow trout	Salmon
Pacific cod	0.1579									
Panga	**0.0016**	0.0904								
Alaska pollock	0.0844	0.7057	0.1676							
Yellowfin sole	**0.0230**	0.3641	0.3641	0.5568						
North-Pacific hake	0.0646	0.6142	0.2039	0.8736	0.6424					
Flounder	0.5586	0.3621	**0.0097**	0.2217	0.0803	0.1797				
Mackerel	0.2620	0.7067	**0.0432**	0.4817	0.2205	0.4090	0.5410			
Rainbow trout	0.2049	0.8438	0.0642	0.5905	0.2884	0.5083	0.4447	0.8360		
Salmon	0.1095	0.8107	0.1333	0.8682	0.4741	0.7621	0.2719	0.5653	0.6843	
Bream	0.0635	0.6058	0.1962	0.8599	0.6400	0.9756	0.1769	0.4021	0.5006	0.7511
**Mn** Fish species	Nile tilapia	Pacific cod	Panga	Alaska pollock	Yellowfin sole	North-Pacific hake	Flounder	Mackerel	Rainbow trout	Salmon
Pacific cod	**0.0004**									
Panga	**0.0039**	0.5127								
Alaska pollock	**0.0000**	0.5333	0.2293							
Yellowfin sole	**0.0001**	0.6506	0.2990	0.8204						
North-Pacific hake	**0.0000**	0.5774	0.2548	0.9210	0.8849					
Flounder	0.7734	**0.0011**	**0.0082**	**0.0001**	**0.0002**	**0.0001**				
Mackerel	**0.0022**	0.6173	0.8399	0.2953	0.3731	0.3241	**0.0051**			
Rainbow trout	**0.0203**	0.2365	0.5414	0.0838	0.1177	0.0959	**0.0364**	0.4478		
Salmon	0.0629	0.1024	0.2865	**0.0280**	**0.0430**	**0.0332**	0.0998	0.2267	0.5983	
Bream	0.0935	0.0662	0.2083	**0.0155**	**0.0253**	**0.0189**	0.1371	0.1594	0.4653	0.7986
**Cr** Fish species	Nile tilapia	Pacific cod	Panga	Alaska pollock	Yellowfin sole	North-Pacific hake	Flounder	Mackerel	Rainbow trout	Salmon
Pacific cod	0.8799									
Panga	0.5692	0.6407								
Alaska pollock	0.9722	0.8948	0.5716							
Yellowfin sole	0.9772	0.8956	0.5776	0.9930						
North-Pacific hake	0.5800	0.6396	0.9741	0.5755	0.5862					
Flounder	0.3902	0.4499	0.7320	0.3933	0.3972	0.7211				
Mackerel	0.4271	0.4862	0.7815	0.4281	0.4336	0.7729	0.9280			
Rainbow trout	**0.0099**	**0.0130**	**0.0395**	**0.0097**	**0.0101**	**0.0396**	0.0728	0.0668		
Salmon	**0.0146**	**0.0186**	0.0519	**0.0141**	**0.0148**	0.0528	0.0887	0.0843	0.8721	
Bream	0.0907	0.1083	0.2249	0.0891	0.0920	0.2267	0.3286	0.3177	0.3573	0.4105
**Cu** Fish species	Nile tilapia	Pacific cod	Panga	Alaska pollock	Yellowfin sole	North-Pacific hake	Flounder	Mackerel	Rainbow trout	Salmon
Pacific cod	0.2130									
Panga	0.2964	0.7273								
Alaska pollock	0.2612	0.8693	0.8319							
Yellowfin sole	0.9180	0.1885	0.2824	0.2338						
North-Pacific hake	0.4943	0.0627	0.1056	0.0829	0.5291					
Flounder	0.2877	0.8047	0.8984	0.9196	0.2605	0.0952				
Mackerel	0.2912	0.7753	0.9342	0.8848	0.2681	0.0986	0.9564			
Rainbow trout	0.1285	0.7378	0.5212	0.6397	0.1106	**0.0314**	0.5864	0.5613		
Salmon	0.1868	0.9043	0.6575	0.7934	0.1632	0.0523	0.7325	0.7033	0.8055	
Bream	0.1898	0.9217	0.6710	0.8066	0.1666	0.0535	0.7456	0.7170	0.7964	0.9754
**Al** Fish species	Nile tilapia	Pacific cod	Panga	Alaska pollock	Yellowfin sole	North-Pacific hake	Flounder	Mackerel	Rainbow trout	Salmon
Pacific cod	**0.0008**									
Panga	**0.0008**	0.9534								
Alaska pollock	0.9697	**0.0007**	**0.0008**							
Yellowfin sole	0.0558	0.1437	0.1353	0.0520						
North-Pacific hake	0.0650	0.1245	0.1239	0.0563	0.8903					
Flounder	**0.0001**	0.6244	0.6021	**0.0001**	0.0632	0.0511				
Mackerel	**0.0003**	0.8034	0.7747	**0.0003**	0.1007	0.0842	0.7823			
Rainbow trout	**0.0000**	0.4592	0.4370	**0.0000**	**0.0368**	**0.0281**	0.7588	0.5904		
Salmon	**0.0001**	0.5077	0.4849	**0.0001**	**0.0440**	**0.0342**	0.8252	0.6466	0.9148	
Bream	**0.0001**	0.5068	0.4865	**0.0001**	**0.0428**	**0.0335**	0.8284	0.6458	0.9056	0.9837
**Li** Fish species	Nile tilapia	Pacific cod	Panga	Alaska pollock	Yellowfin sole	North-Pacific hake	Flounder	Mackerel	Rainbow trout	Salmon
Pacific cod	0.8431									
Panga	0.7130	0.8418								
Alaska pollock	0.5942	0.4953	0.4105							
Yellowfin sole	0.8362	0.9807	0.8492	0.4995						
North-Pacific hake	0.1651	0.1284	0.1001	0.3460	0.1332					
Flounder	0.0818	0.1118	0.1368	**0.0269**	0.1073	**0.0018**				
Mackerel	0.0854	0.1174	0.1499	**0.0282**	0.1150	**0.0019**	0.9928			
Rainbow trout	**0.0291**	**0.0433**	0.0593	**0.0074**	**0.0425**	**0.0003**	0.6222	0.6033		
Salmon	**0.0034**	**0.0058**	**0.0091**	**0.0005**	**0.0057**	**0.0000**	0.2366	0.2212	0.4334	
Bream	0.1915	0.2461	0.2837	0.0776	0.2362	**0.0082**	0.6126	0.6310	0.3528	0.1065
**As** Fish species	Nile tilapia	Pacific cod	Panga	Alaska pollock	Yellowfin sole	North-Pacific hake	Flounder	Mackerel	Rainbow trout	Salmon
Pacific cod	0.2318									
Panga	0.4363	0.0561								
Alaska pollock	0.2822	0.8416	0.0777							
Yellowfin sole	0.6039	0.0997	0.7547	0.1316						
North-Pacific hake	0.3733	0.6736	0.1190	0.7970	0.1907					
Flounder	0.9928	0.2387	0.4217	0.2981	0.5841	0.4005				
Mackerel	**0.0450**	0.3782	**0.0055**	0.3118	**0.0128**	0.2238	**0.0469**			
Rainbow trout	0.1047	0.6161	**0.0181**	0.5219	**0.0369**	0.3964	0.1087	0.6591		
Salmon	0.2236	0.9887	0.0546	0.8410	0.0968	0.6696	0.2336	0.3909	0.6310	
Bream	**0.0062**	0.1174	**0.0004**	0.0911	**0.0011**	0.0574	**0.0066**	0.4360	0.2524	0.1243
**Pb** Fish species	Nile tilapia	Pacific cod	Panga	Alaska pollock	Yellowfin sole	North-Pacific hake	Flounder	Mackerel	Rainbow trout	Salmon
Pacific cod	**0.0009**									
Panga	0.5072	**0.0076**								
Alaska pollock	0.0877	0.1090	0.2533							
Yellowfin sole	**0.0042**	0.6304	**0.0243**	0.2258						
North-Pacific hake	0.0526	0.1741	0.1732	0.7602	0.3246					
Flounder	**0.0007**	0.9072	**0.0060**	0.0935	0.5717	0.1532				
Mackerel	**0.0001**	0.5839	**0.0014**	**0.0375**	0.3346	0.0690	0.6459			
Rainbow trout	**0.0003**	0.7290	**0.0030**	0.0606	0.4410	0.1053	0.8002	0.8080		
Salmon	**0.0006**	0.8597	**0.0052**	0.0860	0.5398	0.1430	0.9416	0.6808	0.8427	
Bream	**0.0012**	0.9184	**0.0091**	0.1213	0.6790	0.1885	0.8384	0.5329	0.6706	0.7943
**Cd** Fish species	Nile tilapia	Pacific cod	Panga	Alaska pollock	Yellowfin sole	North-Pacific hake	Flounder	Mackerel	Rainbow trout	Salmon
Pacific cod	**0.0000**									
Panga	0.5182	**0.0000**								
Alaska pollock	0.2294	**0.0006**	0.0782							
Yellowfin sole	0.2264	**0.0006**	0.0764	0.9522						
North-Pacific hake	0.5533	**0.0000**	0.2451	0.4915	0.4852					
Flounder	**0.0000**	0.9110	**0.0000**	**0.0007**	**0.0007**	**0.0000**				
Mackerel	**0.0000**	0.9156	**0.0000**	**0.0007**	**0.0008**	**0.0001**	0.9887			
Rainbow trout	**0.0000**	0.6835	**0.0000**	**0.0020**	**0.0020**	**0.0002**	0.7363	0.7438		
Salmon	**0.0015**	0.1531	**0.0001**	**0.0430**	**0.0386**	**0.0084**	0.1655	0.1733	0.2628	
Bream	**0.0013**	0.1643	**0.0001**	**0.0426**	**0.0416**	**0.0080**	0.1705	0.1833	0.2628	0.9407
**Hg** Fish species	Nile tilapia	Pacific cod	Panga	Alaska pollock	Yellowfin sole	North-Pacific hake	Flounder	Mackerel	Rainbow trout	Salmon
Pacific cod	0.0539									
Panga	**0.0479**	**0.0001**								
Alaska pollock	0.9665	0.0550	0.0510							
Yellowfin sole	0.6851	**0.0222**	0.0937	0.6767						
North-Pacific hake	0.6420	0.1216	**0.0201**	0.6555	0.4199					
Flounder	**0.0067**	**0.0000**	0.4179	**0.0070**	**0.0174**	**0.0019**				
Mackerel	0.3176	0.3120	**0.0036**	0.3221	0.1813	0.5334	**0.0002**			
Rainbow trout	0.1842	0.4939	**0.0010**	0.1875	0.0950	0.3406	**0.0000**	0.6924		
Salmon	0.6959	0.1095	**0.0234**	0.7058	0.4601	0.9193	**0.0024**	0.4992	0.3141	
Bream	0.0252	0.7220	**0.0000**	0.0260	0.0091	0.0658	**0.0000**	0.1943	0.3304	0.0576
